# Identification of two mutations increasing the methanol tolerance of *Corynebacterium glutamicum*

**DOI:** 10.1186/s12866-015-0558-6

**Published:** 2015-10-16

**Authors:** Lennart Leßmeier, Volker F. Wendisch

**Affiliations:** Genetics of Prokaryotes, Faculty of Biology & Center for Biotechnology, Bielefeld University, Bielefeld, 33615 Germany

**Keywords:** *Corynebacterium*, Methanol, Tolerance, O-acetylhomoserine sulfhydrolase, Coenzyme A hydrolase/transferase, Methionine, Ethanol, Acetyl-CoA, MetY, Cat

## Abstract

**Background:**

Methanol is present in most ecosystems and may also occur in industrial applications, e.g. as an impurity of carbon sources such as technical glycerol. Methanol often inhibits growth of bacteria, thus, methanol tolerance may limit fermentative production processes.

**Results:**

The methanol tolerance of the amino acid producing soil bacterium *Corynebacterium glutamicum* was improved by experimental evolution in the presence of methanol. The resulting strain Tol1 exhibited significantly increased growth rates in the presence of up to 1 M methanol. However, neither transcriptional changes nor increased enzyme activities of the linear methanol oxidation pathway were observed, which was in accordance with the finding that tolerance to the downstream metabolites formaldehyde and formate was not improved. Genome sequence analysis of strain Tol1 revealed two point mutations potentially relevant to enhanced methanol tolerance: one leading to the amino acid exchange A165T of O-acetylhomoserine sulfhydrolase MetY and the other leading to shortened CoA transferase Cat (Q342*). Introduction of either mutation into the genome of *C. glutamicum* wild type increased methanol tolerance and introduction of both mutations into *C. glutamicum* was sufficient to achieve methanol tolerance almost indistinguishable from that of strain Tol1.

**Conclusion:**

The methanol tolerance of *C. glutamicum* can be increased by two point mutations leading to amino acid exchange of O-acetylhomoserine sulfhydrolase MetY and shortened CoA transferase Cat. Introduction of these mutations into producer strains may be helpful when using carbon sources containing methanol as component or impurity.

**Electronic supplementary material:**

The online version of this article (doi:10.1186/s12866-015-0558-6) contains supplementary material, which is available to authorized users.

## Background

Methanol naturally occurs in most ecosystems and is the second most abundant organic gas in the atmosphere besides methane [1]. The major source for methanol is the emission by plants [2]. Also the decay of plants, biomass burning or atmospheric oxidation of methane give rise to methanol [3]. One example of microbial production of methanol in nature is the pectin degradation by *Clostridium butyricum* [4].

Methanol itself is cytotoxic since it affects the fluidity of cellular membranes and alters their mechanical stability [5]. Membrane disruption has been reported for incubation with more than 44 % methanol [6]. Also indirect toxic effects related to methanol have been reported, mostly due to accumulation of the methanol degradation products formaldehyde and formate. Formaldehyde is a potent cytotoxin due to its high reactivity with proteins and DNA [7, 8]. In mammalian species, toxicity of methanol is mainly attributed to an accumulation of formate, causing metabolic acidosis [9]. Formate has also been demonstrated to inhibit mitochondrial cytochrome oxidase of mammals [10, 11]. Additionally, the oxidation of methanol and its metabolites is often accompanied by the generation of superoxide anions, which give rise to oxidative stress and may be involved in lipid peroxidation [12, 13].

According to its high abundance, degradation of methanol is a common feature in nature. Besides detoxification, methanol can also be utilized as a carbon and energy source by a wide variety of eukaryotic and prokaryotic methylotrophs [14]. Metabolism of methanol is typically initiated by its oxidation, which can be catalyzed by many different enzymes such as pyrroloquinoline quinone (PQQ)-dependent methanol dehydrogenase e.g. in *Methylobacterium extorquens* [15], class I alcohol dehydrogenase e.g. in humans [16] or alcohol oxidase e.g. in *Candida boidinii* [17].

The resulting formaldehyde is a branching point between detoxification pathways and the assimilation pathways in methylotrophs. In non-methylotroph organisms, the detoxification of formaldehyde typically occurs in linear pathways, in which formaldehyde is oxidized to formate by formaldehyde dehydrogenase and further to carbon dioxide catalyzed by formate dehydrogenase [14].

The Gram-positive bacterium *Corynebacterium glutamicum* belongs to the mycolic acid-containing actinomycetes and is particularly known for its use in the million-ton-scale production of amino acids [18, 19]. Recent studies on this organism revealed that *C. glutamicum* possesses an endogenous pathway for the oxidation of methanol to carbon dioxide. In this pathway, the oxidation of methanol to formaldehyde is mainly performed by the alcohol dehydrogenase AdhA (cg3107), but at least one additional enzyme of hitherto unknown identity is also involved [20]. Formaldehyde is oxidized by two distinct enzymes, the acetaldehyde dehydrogenase Ald (cg3096) and the mycothiol-dependent formaldehyde dehydrogenase FadH (cg0387) [21]. The resulting formate is subsequently converted to carbon dioxide by formate dehydrogenase FdhF (cg0618) also involving the gene products encoded by cg0616 and cg0617 [22]. The electron acceptor of Fdh is currently unknown.

Methanol tolerance can be a bottleneck in industrial biotechnology if the culture broth contains methanol, either as a part of the process or an impurity e.g. of the carbon source. For example, *Gluconobacter frateurii* needed to be adapted to high methanol concentrations before it could be cultivated using methanol-containing raw glycerol as a substrate, which occurs as a byproduct during biodiesel production [23]. Additionally, methanol represents an interesting upcoming carbon source for microbial production of chemicals [24, 25] e.g. cadaverine [26]. *C. glutamicum* cannot use methanol as sole carbon source [20, 21], although engineered strains do convert methanol to a certain degree to intracellular metabolites [27] and to products such as cadaverine [28].

However, *C. glutamicum* has been engineered to use pure glycerol for growth and amino acid production [29], while certain technical qualities of glycerol obtained from bio-diesel factories, which contain methanol as impurity, were inhibitory [30]. The aim of this study was to improve the methanol tolerance of *C. glutamicum*. Genome sequence analysis of an evolved strain and subsequent genetic and physiological experiments revealed that two single nucleotide polymorphisms (SNPs) significantly increased the tolerance to methanol of *C. glutamicum* without directly affecting the methanol detoxification pathway.

## Results

### Biphasic, non-linear growth response of *C. glutamicum* wild type to methanol

**Fig. 1:**
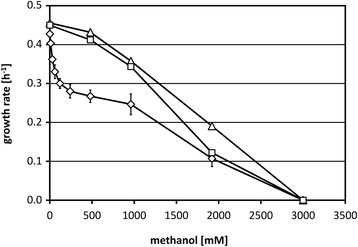
Dependence of the growth rate of *C. glutamicum* WT(pVWEx1) and of two evolved mutant strains on the methanol concentration added to glucose minimal medium. Growth rates of *C. glutamicum* WT(pVWEx1) (*diamonds*) and the mutant strains Tol1 (*triangles*) and Tol2 (*squares*) on minimal medium with 100 mM glucose and varying methanol concentrations

In accordance with operation of linear methanol detoxification in *C. glutamicum* [21, 22], this bacterium is able to grow in the presence of up to 1.3 M methanol reaching high biomass concentrations [20]. When *C. glutamicum* wild-type strain ATCC 13032 carrying the vector pVWEx1 was grown in the presence of a wide range of methanol concentrations, however, the growth rate showed a non-linear dependency on methanol (Fig. 1). In a first concentration range up to 120 mM methanol, a sharp decrease of the growth rate was observed with a growth rate in the presence of 120 mM methanol decreased by 30 % (0.30 ± 0.01 h^-1^) as compared to growth without methanol (0.43 ± 0.00 h^−1^). In the second concentration range from 480 mM to 3 M methanol, the growth rate gradually decreased but with a much smaller slope as e.g. increasing the methanol concentration eight fold from 120 mM to 960 mM only reduced the growth rate from 0.30 ± 0.01 h^-1^ to 0.25 ± 0.03 h^−1^ (Fig. 1). The bi-phasic, non-linear dependence of the growth rate on the presence of methanol may indicate that the effect of methanol on growth of *C. glutamicum* may be more complex than anticipated.

### Adaptive laboratory evolution of *C. glutamicum* in the presence of methanol

Experimental evolution in selective medium was used to achieve a genetic adaption to the presence of methanol and thereby increasing the methanol tolerance of *C. glutamicum*. For this purpose, repeated cultivations with the wild-type strain ATCC 13032 carrying the vector pVWEx1 were performed in minimal medium containing 120 mM methanol. After this selection process, growth of two independently isolated strains, named Tol1 and Tol2, was analyzed in the presence and absence of methanol. In the absence of methanol, strains Tol1 and Tol2 and the parental strain *C. glutamicum* (pVWEx1) showed similar growth behavior with a growth rate of 0.44 ± 0.01 h^-1^ in glucose minimal medium. A comparison of the growth behavior at diverse methanol concentrations revealed that the methanol tolerant strains showed significantly increased growth rates at concentrations up to 1 M, while growth in the presence of 3 M methanol was neither observed for the evolved strains nor for the parental strain (Fig. 1). Interestingly, unlike the wild type, the dependence of the growth rate on methanol was almost linear for the tolerant strains (Fig. 1), and lower methanol concentrations did not cause severe growth retardation of the tolerant strains. Because both mutant strains showed a similar phenotype, only Tol1 was used in further analyses to identify the mutation(s) overcoming the strong growth impairment by low methanol concentrations.

### Role of the linear methanol detoxification pathway

*C. glutamicum* possesses the linear methanol detoxification pathway involving oxidation of methanol via formaldehyde and formate to carbon dioxide. In order to test whether methanol oxidation by Tol1 differs from *C. glutamicum* wild type, enzyme activity of alcohol dehydrogenase AdhA was determined. The specific alcohol dehydrogenase activities with ethanol as substrate were comparable for *C. glutamicum* wild type (79 ± 2 mU/mg) and Tol1 (78 ± 1 mU/mg) grown in LB. Under inducing conditions [20, 31, 32], i.e. after growth in LB with 325 mM ethanol, alcohol dehydrogenase activities increased in *C. glutamicum* wild type (183 ± 18 mU/mg) and Tol1 (276 ± 27 mU/mg). Moreover, as growth of an *adhA* deletion mutant in glucose minimal medium with up to 960 mM methanol was comparable to that of *C. glutamicum* wild type (data not shown), AdhA did not contribute notably to the response of *C. glutamicum* to methanol.

Methanol oxidation gives rise to the highly toxic metabolite formaldehyde. In glucose minimal medium without formaldehyde, the growth rates were comparable for *C. glutamicum* WT(pVWEx1) (0.41 ± 0.01 h^−1^) and Tol1 (0.42 ± 0.01 h^−1^) and they were decreased similarly to 0.32 ± 0.01 h^−1^, when formaldehyde was present (Additional file 1: Fig. S1a). In addition, the specific activities of formaldehyde oxidizing enzymes Ald and FadH determined by an in vivo assay (Additional file 1: Figure S1b) were comparable for both strains (22 ± 0 and 23 ± 1 nmol min^−1^ mg cell dry weight^−1^ for Tol1 and WT(pVWEx1), respectively). In addition, growth of *C. glutamicum* Tol1 and WT(pVWEx1) in the presence of 200 mM potassium formate, the second potentially toxic intermediate of the linear methanol oxidation pathway, was comparable (each 0.24 ± 0.01 h^−1^, data not shown). Thus, *C. glutamicum* Tol1 showed improved tolerance to methanol, but neither to formaldehyde nor to formate.

### DNA microarray analysis of global gene expression of *C. glutamicum* Tol1

Genome-wide gene expression analyses using microarrays were performed in order to identify differentially expressed genes in strain Tol1, which might contribute to methanol tolerance. In a first experiment, mRNA levels of Tol1 and *C. glutamicum* WT(pVWEx1) were compared during exponential growth in complex medium. The genes *cysK* and *metY* which are involved in amino acid metabolism and the *prpD2B2C2* operon showed higher mRNA levels in Tol1 than in the parental strain (Additional file 1: Table S1). The *prpD2B2C2* operon which codes for enzymes required for propionate metabolism shows a strong induction in presence of propionate [33]. To analyze if the increased expression of this operon positively affects growth with methanol, *C. glutamicum* wild type was grown in glucose minimal medium with 0 mM or 7 mM propionate and 120 mM methanol was added after two hours. Methanol decreased the growth rate by about 45 % in the presence or absence of propionate (Additional file 1: Fig. S2), demonstrating that the induction of the *prpD2B2C2* operon did not increase the tolerance to methanol.

**Fig. 2:**
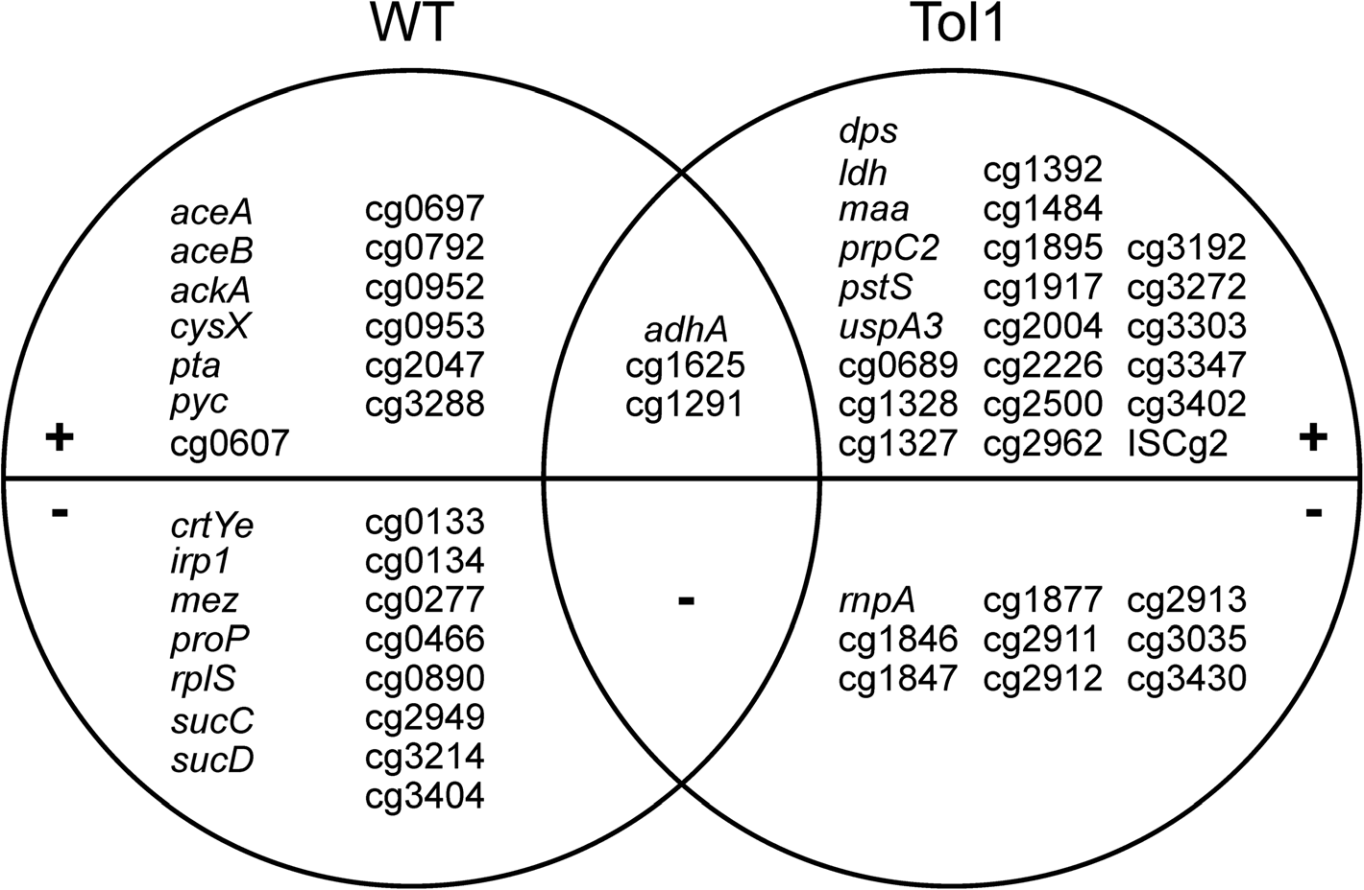
Venn diagram showing gene expression changes in a comparison of *C. glutamicum* Tol1 with wild type. The strains were cultivated in minimal medium with 100 mM glucose in the presence or absence of methanol. Genes with increased mRNA levels in the presence of methanol are shown in the (+) section and genes with reduced mRNA levels are shown in the (−) section of the graph

In a second experiment, global gene expression changes of *C. glutamicum* Tol1 and WT due to addition of methanol were compared. *C. glutamicum* WT was cultivated in glucose minimal medium with/without addition of 30 mM methanol. To avoid growth rate-dependent differences, the methanol tolerant strain Tol1 was cultivated in the presence of 120 mM methanol, a methanol concentration leading to a comparable growth rate reduction. As consequence of methanol addition, expression of 35 and 31 genes, respectively, changed in *C. glutamicum* Tol1 and WT, respectively (Additional file 1: Table S2). Expression of only three genes, namely *adhA* and two genes for hypothetical proteins (cg1625 and cg1291; Fig. 2), changed in both strains. It was observed that expression of genes responsible for ethanol utilization via acetate in the glyoxylate cycle (*adhA, aceA* for isocitrate lyase, *aceB* for malate synthase, *ackA* for acetate kinase, *pta* for phosphate acetyltransferase and *sucCD* for succinyl-CoA synthetase) were induced in the wild type, but not in Tol1. Since expression of these genes is activated in acetate minimal medium by transcriptional activator RamA [34–36], growth of *C. glutamicum* Δ*ramA* in the presence of methanol was analyzed (Additional file 1: Fig. S3). However, growth of *C. glutamicum* Δ*ramA* and of the wild type in glucose minimal medium was similar without methanol (0.36 ± 0.00 h^−1^ vs. 0.39 ± 0.00 h^−1^) and with 480 mM methanol (0.23 ± 0.00 h^−1^ vs. 0.25 ± 0.00 h^−1^).

### Genome sequencing of Tol1 and introduction of mutations into the wild-type genome

Since neither physiological experiments and enzyme activity measurements nor DNA microarray analysis revealed the mutation(s) responsible for enhanced methanol tolerance of *C. glutamicum* Tol1, its genome was sequenced. Compared to the published genome sequence of *C. glutamicum* ATCC 13032 [37], 101 insertions, deletions or SNPs were found (Additional file 1: Table S3). Previously, a similar number of changes has been observed when genomes of *C. glutamicum* mutants were sequenced [38, 39]. Therefore, all sequence changes found in the genome of Tol1 were compared to the genome sequence of a control strain derived from the same wild type during the same period of time. Only 29 mutations were unique for Tol1. Of these, one SNP representing a synonymous substitution, four SNPs located in intergenic regions and 19 SNPs in a gene (cg2069) a putative secreted protein of the prophage CGP3 [37] were not considered further.

**Fig. 3:**
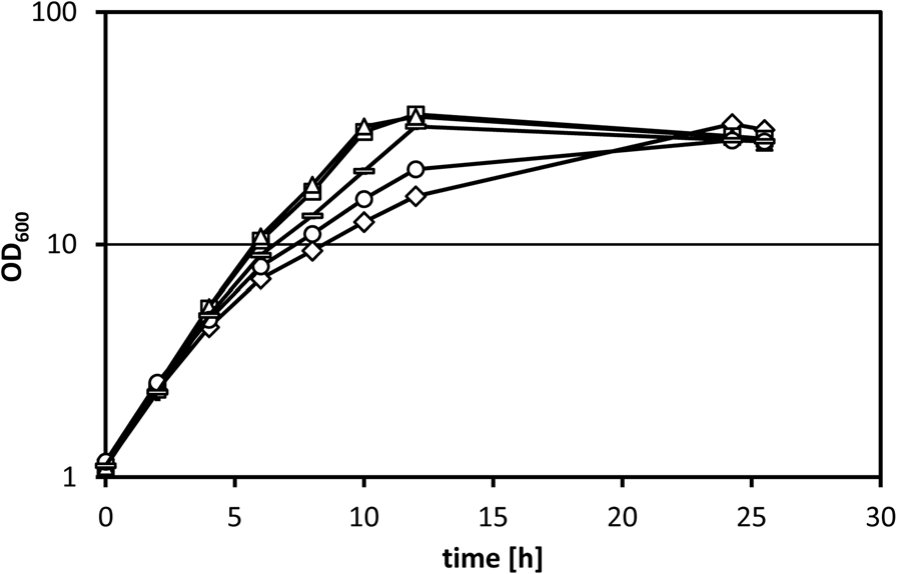
Growth of various *C. glutamicum* strains in glucose minimal medium supplemented with 240 mM methanol. Growth of *C. glutamicum* WT (*diamonds*), Tol1 (*triangles*), T0755 (*circles*), T2840 (*bars*) and T0755 + 2840 (*squares*) in minimal medium supplemented with 100 mM glucose and 240 mM methanol. Means and standard deviations of two independent cultures are shown

The remaining five SNPs led to amino acid substitutions: a change of alanine to valine at position 19 (A19V) of conserved hypothetical protein encoded by cg0198, change L328S in the putative membrane protein encoded by cg1245, change D67H in the ABC-type transporter subunit encoded by cg2204 and change A165T in the O-acetylhomoserine sulfhydrolase MetY. One SNP caused a nonsense mutation (Q342*) and resulted in a truncated version of CoA transferase Cat lacking the 161 C-terminal amino acids. These five mutations were introduced individually into the genome of *C. glutamicum* WT resulting in the strains T0198, T0755, T1245, T2204 and T2840. None of these mutations affected growth in glucose minimal medium or in complex medium (data not shown). Growth of strains T0198, T1245 and T2204 in glucose minimal medium supplemented with 240 mM methanol was comparable to that of the wild type (data not shown). However, strain T0755 showed a slightly increased growth rate (0.32 ± 0.00 h^−1^) in glucose minimal medium supplemented with 240 mM methanol as compared to WT (0.31 ± 0.00 h^−1^), while strain T2840 grew significantly faster (0.35 ± 0.00 h^−1^) (Fig. 3). The mutation of *cat* observed in Tol1 caused a truncation of the enzyme at position 342, thus, while the N-terminal acetyl-CoA hydrolase/transferase domain (pfam02550) predicted by alignment to the conserved domain database (CDD) [40] is present, the respective C-terminal domain (pfam13336) is not. This severe modification may have changed its enzymatic (side) activity or caused a loss of enzyme function. To analyze if the latter causes the increased methanol tolerance, a *cat* deletion strain lacking both the N- and C-terminal regions of Cat [41] was analyzed. In the absence of methanol, this strain showed a slightly lower growth rate (0.38 ± 0.00 h^−1^) than WT (0.39 ± 0.00 h^−1^), while the growth rate of Δ*cat* (0.29 ± 0.00 h^−1^) was significantly higher than that of the WT (0.20 ± 0.00 h^−1^) in minimal medium with 100 mM glucose and 240 mM methanol (Additional file 1: Fig. S4). These results indicated that the lack of Cat activity improved methanol tolerance to a comparable extent as observed when Cat was truncated due to the nonsense mutation (Q342*).

When both mutations were combined, the resulting strain T0755 + 2840 grew as fast (0.38 ± 0.00 h^−1^) as Tol1 in glucose minimal medium supplemented with 240 mM methanol (Fig. 3). Thus, the identified two mutations resulting in amino acid exchange A165T in the O-acetylhomoserine sulfhydrolase MetY and in truncation (Q342*) of CoA transferase were sufficient to explain the improved methanol tolerance of Tol1.

### Influence of mutations increasing methanol tolerance on growth with ethanol

**Fig. 4:**
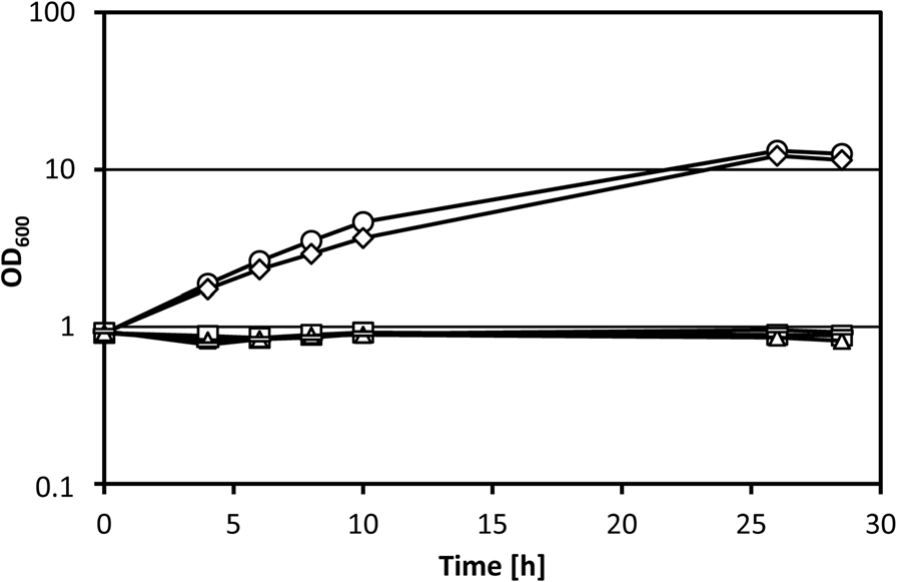
Growth of *C. glutamicum* mutants with ethanol. Growth of *C. glutamicum* WT (*diamonds*), Tol1 (*triangles*), T0755 (*circles*), T2840 (*bars*) and T0755 + 2840 (*squares*) in minimal medium supplemented with 1.5 % ethanol as sole source of carbon and energy. Means and standard deviations of two independent cultures are shown

*C. glutamicum* wild type is able to utilize ethanol, but not methanol, as sole carbon source [31]. To determine if one or both of the mutations increasing tolerance to methanol affects growth with ethanol as sole carbon source, growth experiments with *C. glutamicum* WT, Tol1, T0755, T2840 and T0755 + 2840 in minimal medium containing 325 mM ethanol as sole carbon source were performed. Surprisingly, strain Tol1 showed no growth on ethanol, while *C. glutamicum* WT grew with a growth rate of 0.14 ± 0.00 h^−1^ (Fig. 4). Strain T0755 grew with ethanol at a growth rate of 0.16 ± 0.00 h^−1^, but neither strain T2840 nor strain T0755 + 2840 were able to grow in ethanol minimal medium (Fig. 4). Thus, truncation of CoA transferase Cat due to missense mutation Q342* as present in strains Tol1, T2840 and T0755 + 2840 resulted in the inability to utilize ethanol as sole source of carbon and energy. To test if a *cat* deletion mutant lacking both the N- and C-terminal parts of Cat is able to grow, *C. glutamicum* WT, Tol1, T2840 and Δ*cat* was grown in minimal medium with 1 % ethanol as sole carbon source. While *C. glutamicum* WT could grow, strains Tol1, T2840 and Δ*cat* did not (Additional file 1: Fig. S5).

## Discussion

In this study, the response of *C. glutamicum* to methanol was characterized to be non-linear involving a sharp decrease of the growth rate in the presence of lower methanol concentrations and a less pronounced decrease evident at methanol concentrations in excess of about 250 mM. Genome sequencing of a strain selected by adaptive laboratory evolution identified two SNPs that subsequently were shown to be sufficient to explain increased methanol tolerance of the selected strain. The relevant SNPs led to amino acid substitution A165T in the O-acetylhomoserine sulfhydrolase MetY and in truncation (Q342*) of CoA transferase by 161 amino acids.

Mutations which increase the tolerance to methanol in other organisms are so far reported to be mostly related to enzymes of methanol pathways and thereby reducing the accumulation of toxic downstream metabolites. For example the methanol tolerance of *Dictyostelium* increased significantly by loss of a catalase, which is supposed to be the main enzyme in this organism oxidizing methanol to formaldehyde [42]. Also methanol tolerance of the methylotroph bacterium *Bacillus methanolicus* is dependent on the activities of the methanol dehydrogenase Mdh and the enzymes 3-hexulose 6-phosphate synthase (Hps) and 6-phospho-3-hexuloisomerase (Phi), responsible for utilization of formaldehyde [43]. However, in *C. glutamicum* deletion of *adhA* did not affect methanol tolerance although the encoded alcohol dehydrogenase oxidizes methanol to formaldehyde. Moreover, the evolved strain Tol1 did not show increased tolerance to the methanol metabolites formaldehyde and formate. Notably, growth of Tol1 was not impaired as drastic as *C. glutamicum* WT at methanol concentrations up to 120 mM, while the maximal methanol concentration tolerated was similar for WT and Tol1. This argued that the sharp decline of the growth rate observed for *C. glutamicum* WT may be caused by methanol itself or compounds derived from methanol in reactions other than those of the linear methanol oxidation pathway. Direct toxic effects of methanol are known to be based on its hydrophobic character, which is affecting the stability of the cellular membrane [5]. It has been shown that the tolerance to other membrane affecting alcohols like butanol could be increased by mutations in genes corresponding to membrane stability [44]. However, the identified mutations increasing methanol tolerance in *C. glutamicum* were not found in genes affecting membrane stability. The second component of the response of *C. glutamicum* to methanol evident at higher methanol concentrations may be due to membrane damages by methanol or toxicity of formate and formaldehyde.

The finding that mutations affecting O-acetylhomoserine sulfhydrolase MetY and CoA transferase Cat were sufficient to explain increased methanol tolerance of *C. glutamicum* Tol1 indicated that their enzymatic (side) reactions contribute to methanol toxicity and are prominent in particular at concentrations up to about 250 mM. In *C. glutamicum*, methionine functions as methyl donor and is synthesized either by transsulfuration or by direct sulfhydrylation catalyzed by MetY [45]. In the reaction of MetY, O-acetylhomoserine is directly converted to homocysteine using sulfide [46]. In addition, MetY is also able to convert O-acetylhomoserine to methionine using methanethiol (Reaction: O-acetyl-homoserine + methanethiol < = > methionine + acetate) [47]. Methanethiol is the thiol equivalent of methanol and it has been shown that MetY from *Corynebacterium acetophilum* and *Saccharomyces cerevisiae* accepts methanol and other short-chain alcohols as substrates in addition to sulfide and methanethiol [48]. The MetY-catalyzed alkylation of O-acetylhomoserine with methanol yields O-methylhomoserine and acetate (Reaction: O-acetyl-homoserine + methanol < = > O-methyl-homoserine + acetate). Since O-methylhomoserine is known to inhibit growth of *E. coli* and other microorganisms [49], this MetY-catalyzed reaction may contribute to the methanol toxicity of *C. glutamicum*. Strain Tol1 synthesized a variant of MetY (A165T) and showed higher RNA levels of *metY* in complex medium with added methanol than *C. glutamicum* WT (Additional file 1: Table S1). Albeit strain Tol1 was not further investigated since O-acetylhomoserine could not be obtained, the location of A165 close to a substrate-cofactor binding motif predicted by CDD-alignment [40] may indicate that binding of the cofactor pyridoxal-5-phosphate, which is essential for the function of MetY [46], and MetY activity are affected. While MetY clearly contributed to methanol toxicity in *C. glutamicum*, the biochemical mechanism remains to be explored by combining in vitro and in vivo approaches such as metabolomics and structure-function analyses of MetY and MetY^A165T^.

The second mutation contributing to increased methanol tolerance of strain Tol1 led to truncation of CoA transferase Cat. Cat transfers CoA between acetyl-, propionyl- and succinyl-CoA thioesters and the respective free acids [41]. Whereas it is known that *cat* is highly and constitutively expressed [41], its function remains elusive since only a role in acetate and propionate catabolism in the absence of acetate kinase Ack and phosphotransacetylase Pta during co-consumption with glucose was found [41, 50]. Both the lack of Cat activity due to deletion of *cat* as well as its truncation due to the SNP present in strain Tol1 increased the methanol tolerance. Thus, either the activity of CoA transfer between the acids acetate, propionate or succinate and the respective thioesters [41], or enzymatic side activity of Cat result in reduced growth in the presence of methanol. Several side reactions appear possible and although not all of them have been documented, in other organisms CoA transferases may have activity as acetyl-CoA hydrolases, as alcohol acetyltransferases or may form methyl-CoA. Alcohol acetyltransferases e.g. from *Saccharomyces uvarum* catalyze the transfer of the acetyl moiety from acetyl-CoA to methanol resulting in methyl acetate ester [51]. Methyl-Coenzyme M is an intermediate in methanogenic archaea like *Methanosarcina barkeri* and is formed by methanol:coenzyme M methyltransferase [52]. Due to structural similarities of the coenzymes A and M, an analogous reaction of methanol with coenzyme A might be possible. Future metabolomics and structure-function analyses are necessary to determine if Cat from *C. glutamicum* WT, but not from Tol1, also possesses activity as acetyl-CoA hydrolase, alcohol acetyltransferase or for generation of methyl-CoA and if the resulting intermediates are growth inhibitory. This also pertains to the finding that truncation of Cat as well as the absence of Cat precluded use of ethanol as sole source of carbon and energy. It is not clear whether increased methanol tolerance and the inability to use ethanol are interdependent or arose by chance as consequence of Cat truncation.

Growth of *C. glutamicum* with ethanol involves oxidation of ethanol to acetate and requires activation by the Pta/Ack-system and operation of the glyoxylate cycle [31]. Thus, acetyl-CoA is an essential intermediate of ethanol metabolism and its concentration would be reduced if truncated Cat were active as acetyl-CoA hydrolase. Alternatively, induction of genes *ack*, *pta*, *aceA* and *aceB* by ethanol [31], which is as high as induction by acetate [50, 53] or methanol [20] may be impaired by truncated Cat. Indeed, induction of these genes was not observed in Tol1 (Additional file 1: Table S2). The genes *ack*, *pta*, *aceA* are directly repressed by RamB [54, 55] and activated by RamA [56, 57]. *AceA* and *aceB* are directly repressed by GlxR, but regulation of the *pta-ack* operon by GlxR has not yet been demonstrated in vivo. GlxR and SugR indirectly control these genes by regulation of *ramA* expression [56]. *C. glutamicum* mutants lacking RamA cannot grow with acetate or ethanol as sole carbon sources [55]. The physiological trigger for the regulation of the ethanol and acetate metabolism is still unknown, but has been inferred as acetyl-CoA or a derivative thereof based on missing induction of genes from the acetate metabolism as result of interrupted *ack* and *pta* genes [53]. Thus, the inducer may not be synthesized or may be degraded by truncated Cat.

The identified mutations increasing methanol tolerance of *C. glutamicum* are relevant to strain development for biotechnological applications either using methanol as (co-)substrate or using growth substrates containing methanol as impurity. The latter was already shown to be of biotechnological relevance since *C. glutamicum* engineered to utilize glycerol for growth and amino acid production readily used pure glycerol, but not all technical qualities of glycerol [29, 30]. Crude glycerol is a by-product of biodiesel production by transesterification of plant fats with methanol and often contains residual methanol [58]. Producer strains carrying the mutations of *metY* and *cat* may show improved performance in processes based on crude glycerol.

## Conclusions

A *C. glutamicum* strain with increased methanol tolerance was selected by adaptive laboratory evolution. Genome sequencing of this strain identified two SNPs leading to amino acid substitution A165T of the O-acetylhomoserine sulfhydrolase MetY and truncation (Q342*) of CoA transferase by 161 amino acids. Introduction of these mutations into the wild type improved tolerance to methanol to the same level as observed with the selected mutant Tol1. Thus, these two mutations were sufficient to explain increased methanol tolerance of the selected strain. Introduction of these mutations into producer strains may facilitate production processes when using methanol as (co-)substrate or using growth substrates containing methanol as impurity.

## Methods

### Microorganisms and cultivation conditions

The strains and plasmids used in this study are listed in Table 1. The *E. coli* strain DH5α was used as a standard cloning host [59].

**Table 1:** Strains and plasmids used in this study

Strain or plasmid	Relevant characteristics	Reference or source
*E. coli* DH5α	F^−^ *thi-1 endA1 hsdR17*(r^−^m^−^) *supE44* Δ*lacU169* (Φ80*lacZ*ΔM15) *recA1 gyrA96 relA1*	[59]
*C. glutamicum* strains		
WT	Wild-type strain ATCC 13032	American Type Culture Collection
Tol1	Methanol tolerant strain derived from *C. glutamicum* WT carrying the vector pVWEx1	This study
Tol2	Methanol tolerant strain derived from *C. glutamicum* WT carrying the vector pVWEx1	This study
Δ*adhA*	in-frame deletion of the *adhA* gene (cg3107) of *C. glutamicum* WT	[21]
Δ*ramA*	in-frame disruption of the *ramA* gene (cg2831) of *C. glutamicum* WT	[55]
T0198	point mutation cg0198^A19V^ from strain Tol1 in the cg0198 gene of *C. glutamicum* WT	This study
T0755	point mutation cg0755^A165T^ from strain Tol1 in the cg0755 gene of *C. glutamicum* WT	This study
T1245	point mutation cg1245^L328S^ from strain Tol1 in the cg1245 gene of *C. glutamicum* WT	This study
T2204	point mutation cg2204^D67H^ from strain Tol1 in the cg2204 gene of *C. glutamicum* WT	This study
T2840	point mutation cg2840^Q342*^ from strain Tol1 in the cg2840 gene of *C. glutamicum* WT	This study
T0755 + 2840	Combination of the point mutations from strain Tol1 in the cg0755 and cg2840 genes of *C. glutamicum* WT	This study
Δ*cat*	in-frame deletion of the *cat* gene (cg2840) of *C. glutamicum* WT lacking both the N- and C-terminal regions of Cat	[41]
Plasmids		
pK19*mobsacB*	Km^r^, mobilizable *E. coli* vector for the construction of insertion and deletion mutants of *C. glutamicum* (*oriV*, *sacB*, *lacZ*α)	[65]
pK19*mobsacB*-T0198	Km^r^, pK19*mobsacB* with the construct for a base exchange from C to T at position 56 in cg0198	This study
pK19*mobsacB*-T0755	Km^r^, pK19*mobsacB* with the construct for a base exchange from G to A at position 493 in cg0755	This study
pK19*mobsacB*-T1245	Km^r^, pK19*mobsacB* with the construct for a base exchange from T to C at position 983 in cg1245	This study
pK19*mobsacB*-T2204	Km^r^, pK19*mobsacB* with the construct for a base exchange from G to C at position 199 in cg2204	This study
pK19*mobsacB*-T2840	Km^r^, pK19*mobsacB* with the construct for a base exchange from C to T at position 1024 in cg2840	This study
pVWEx1	Km^r^; *C. glutamicum*/*E. coli* shuttle vector (*P*_*tac*_ *lacI* ^*q*^ *oriV*_*C.g*_ *. oriV*_*E.c*_.)	[71]

Cultivation of *E. coli* strains was carried out in Luria-Bertani broth complex medium (LB) aerobically on a rotary shaker (120 rpm) at 37 °C. *C. glutamicum* was cultivated aerobically on a rotary shaker (120 rpm) at 30 °C. Growth experiments with *C. glutamicum* were also performed in the microbioreactor system BioLector (m2p labs; Baesweiler, Germany) using FlowerPlate microtiter plates (m2p labs; Baesweiler, Germany). The growth conditions were set to 1100 rpm, 30 °C, 85 % humidity and backscatter gain 20.. LB medium supplemented with 50 mM glucose or bovine heart infusion (BHI) medium were used for precultures. Growth experiments with *C. glutamicum* were performed in the minimal medium mCGXII [21], a modified CGXII medium [60]. The medium was supplemented with 100 mM glucose as carbon and energy source. For selection of clones carrying the plasmids pVWEx1 or pK19*mobsacB* and their derivatives, kanamycin was added to the medium in a concentration of 25 μg ml^−1^.

### Adaptive laboratory evolution

In order to obtain methanol tolerant mutants, *C. glutamicum* (pVWEx1) was repeatedly cultivated in selective mCGXII medium containing 100 mM glucose, kanamycin and 120 mM methanol using two independent cultures. This is the highest concentration in the first concentration range, in which a sharp decrease of the growth rate was observed. The cultures were serially passed to fresh medium in regular intervals for about 50 generations.

The cultures grown in selective medium were subsequently cultivated on BHI plates with kanamycin. Single colonies from these plates were again cultivated in BHI Kan_25_ liquid medium followed by analysis of the methanol tolerance to exclude non-genetic adaption to methanol.

### DNA preparation, manipulation and transformation

Plasmid isolation, molecular cloning and transformation of *E. coli* as well as electrophoresis were performed using standard procedures [61]. Transformation of *C. glutamicum* was performed by electroporation as described previously [62]. Chromosomal DNA of *C. glutamicum* was prepared as formerly described [63]. PCR experiments were performed using GoTaq DNA polymerase (Promega) or KOD Hot Start polymerase (Novagen) with oligonucleotides obtained from Metabion (listed in Additional file 1: Table S4). All restriction enzymes and polynucleotide kinase were obtained from Fermentas and used according to the manufacturer’s instructions. Dephosphorylation and ligation were performed using the Rapid DNA Dephos & Ligation Kit from Roche. Plasmids were isolated from *E. coli* using the QIAprep miniprep kit (Qiagen, Hilden, Germany). DNA sequencing was used to control all inserts of the plasmid constructs listed in Table 1.

### Construction of *C. glutamicum* mutant strains

The single base substitutions in cg0198, cg0755, cg1245, cg2204 and cg2840 were constructed in *C. glutamicum* WT using the corresponding derivatives of plasmid pK19*mobsacB* as described previously [64]. These vectors carry the mutated basepairs flanked by native sequences upstream and downstream. The flanking regions were amplified from genomic DNA of *C. glutamicum* via PCR using corresponding primer pairs A and B as well as C and D (Additional file 1: Table S4). The resulting PCR products as well as primers A and D were used in the subsequent crossover PCR reaction. The resulting fusion product was phosphorylated with polynucleotide kinase and ligated into *Sma*I digested vector pK19*mobsacB* [65]*.* Point mutations were verified by amplification using the primers A and D and subsequent sequence analysis.

### Alcohol dehydrogenase enzyme assays

Enzyme activity of AdhA was measured in crude cell extracts at 30 °C using a Shimadzu UV-1800 spectrophotometer by following the formation of NADH at 340 nm (Δε = 6230 M^−1^ cm^−1^). Crude cell extracts were prepared using sonication treatment [21]. Buffers and assay conditions have been described previously [31] and the reaction was started by addition of 1 M ethanol.

### Analysis of formaldehyde degradation

In vivo formaldehyde degradation assays were performed using resting cells. For this purpose, 50 ml mCGXII medium without carbon source was inoculated from a LB culture to an OD_600_ of 1 and incubated in a 500 ml baffled Erlenmeyer flask at 30 °C and 120 rpm. The assays were started by addition of 0.5 mM formaldehyde. Measurement of formaldehyde concentrations was performed using a colorimetric method as described previously [21].

### Transcriptome analysis using DNA microarrays

Gene expression analysis in *C. glutamicum* WT and strain Tol1 was performed after cultivation in LB or mCGXII medium. RNA was isolated during the mid log growth phase followed by synthesis of fluorescently labeled cDNA from RNA, DNA microarray hybridization and gene expression analysis [66, 67]. The data were normalized using the LOWESS approach. The significance of gene expression rates was determined using a *t*-test adjusted with the False Discovery Rate approach. Individual data points were not considered as significant if the signal to noise ratio of both channels was below 3, less than two third of the replicates showed regulation or the A-value was below 8. Furthermore, the adjusted p-value had to be higher than 0.05 and the genes needed to be regulated more than two-fold.

### Genome sequence analysis

Libraries were prepared from isolated genomic DNA as described previously [68]. Sequencing of the libraries was performed on the Genome Analyze *II*x platform (Illumina, San Diego, CA, USA) using a single read cluster generation kit v4 according to the manufacturer’s instructions. 32 bp sequence reads were mapped to the genome sequence of *C. glutamicum* ATCC13032 [37] using the program SARUMAN [69]. The coverage was obtained by multiplying the read length by the respective read start. Perl programming language script implemented for Parsing of the read start information and calculation of coverage and read start numbers. Variants were considered to be significant if they possessed a frequency higher than 90 % and coverage of at least 70.

## Availability of supporting data

All supporting data are included as additional files. Microarray data have also been deposited in NCBI's Gene Expression Omnibus [70] and are accessible through GEO Series accession number GSE71590 (http://www.ncbi.nlm.nih.gov/geo/query/acc.cgi?acc=GSE71590).

## Additional file

Additional file 1: Table S1. Genome-wide comparison of mRNA levels between the strains Tol1 and *C. glutamicum* WT(pVWEx1) during growth in LB complex medium. **Table S2.** Genome-wide comparison of mRNA levels in Tol1 and *C. glutamicum* wild type cultivated in minimal medium with 100 mM glucose in presence or absence of methanol. **Table S3.** Mutations detected in strain Tol1 and a control strain in comparison to the *C. glutamicum* wild type genome sequence NC_006958.1. **Table S4.** Oligonucleotides used in this study. **Figure S1.** Growth in the presence of formaldehyde (A) and formaldehyde degradation (B). **Figure S2.** Growth of *C. glutamicum* wild type with propionate and methanol. **Figure S3**. Growth of *C. glutamicum* Δ*ramA* with methanol. **Figure S4.** Growth of *C. glutamicum* Δ*cat* with methanol. Figure S5. Biomass formation of *C. glutamicum* wild type, Tol1, T2840 and Δ*cat* in minimal medium with ethanol as sole carbon source. (PDF 919 kb)

## Abbreviations

PQQ: Pyrroloquinoline quinone
AdhA: Alcohol dehydrogenase
Ald: Acetaldehyde dehydrogenase
FadH: Mycothiol-dependent formaldehyde dehydrogenase
SNP: Single nucleotide polymorphism
CoA: Coenzyme A
CDD: Conserved domain database
