# Supplementary material for: Identification of two mutations increasing the methanol tolerance of Corynebacterium glutamicum
Source: BMC Microbiol. 2015 Oct 16;15:216. doi: 10.1186/s12866-015-0558-6 (PMC4609165; doi:10.1186/s12866-015-0558-6)
Supplement: Additional file 1: Table S1. — Genome-wide comparison of mRNA levels between the strains Tol1 and C. glutamicum WT(pVWEx1) during growth in LB complex medium. Table S2. Genome-wide comparison of mRNA levels in Tol1 and C. glutamicum wild type cultivated in minimal medium with 100 mM glucose in presence or absence of methanol. Table S3. Mutations detected in strain Tol1 and a control strain in comparison to the C. glutamicum wild type genome sequence NC_006958.1. Table S4. Oligonucleotides used in this study. Figure S1. Growth in the presence of formaldehyde (A) and formaldehyde degradation (B). Figure S2. Growth of C. glutamicum wild type with propionate and methanol. Figure S3. Growth of C. glutamicum ΔramA with methanol. Figure S4. Growth of C. glutamicum Δcat with methanol. Figure S5. Biomass formation of C. glutamicum wild type, Tol1, T2840 and Δcat in minimal medium with ethanol as sole carbon source. (PDF 919 kb) [file 12866_2015_558_MOESM1_ESM.pdf]

Identification of two mutations increasing the methanol tolerance of *Corynebacterium glutamicum*

Supplementary Information

Lennart Leßmeier & Volker F. Wendisch

Genetics of Prokaryotes, Faculty of Biology & Center for Biotechnology, Bielefeld University, Bielefeld 33615, Germany

Corresponding author:

Prof. Dr. Volker F. Wendisch, Genetics of Prokaryotes, Faculty of Biology & Center for Biotechnology, Bielefeld University, Universitätsstr. 25, 33615 Bielefeld, Germany

volker.wendisch@uni-bielefeld.de

Content:

Table S1 Genome-wide comparison of mRNA levels between the strains Tol1 and *C. glutamicum* WT(pVWEx1) during growth in LB complex medium

Table S2 Genome-wide comparison of mRNA levels in Tol1 and *C. glutamicum* wild type cultivated in minimal medium with 100 mM glucose in presence or absence of methanol

Table S3. Mutations detected in strain Tol1 and a control strain in comparison to the *C. glutamicum* wild type genome sequence NC_006958.1

Table S4 Oligonucleotides used in this study

Figure S1 Growth in the presence of formaldehyde (A) and formaldehyde degradation (B)

Figure S2 Growth of *C. glutamicum* wild type with propionate and methanol

Figure S3. Growth of *C. glutamicum* Δ*ramA* with methanol

Figure S4. Growth of *C. glutamicum* Δ*cat* with methanol.

Figure S5. Biomass formation of *C. glutamicum* wild type, Tol1, T2840 and Δ*cat* in minimal medium with ethanol as sole carbon source.

Supplementary tables

Table S1. Genome-wide comparison of mRNA levels between the strains Tol1 and *C. glutamicum* WT(pVWEx1) during growth in LB complex medium.

Gene ID	Gene name	Annotation	p adjusted [a)]	M-value Tol1/WT [b)]
cg0760	*prpB2*	Methylisocitrate lyase	0.000	1.7
cg0759	*prpD2*	2-Methylcitrate dehydratase	0.000	1.5
cg2833	*cysK*	O-Acetylserine (Thiol)-Lyase	0.000	1.4
cg0762	*prpC2*	2-Methylcitrate synthase	0.031	1.2
cg0755	*metY*	O-Acetylhomoserine sulfhydrylase	0.002	1.1

[a)] p-value adjusted with the False Discovery Rate approach. Genes with a value < 0.05 are considered as significant.
[b)] Dual logarithm of the average ratio of medians. Only genes regulated more than two-fold were considered.
Two biological replicates were performed.

Table S2. Genome-wide comparison of mRNA levels in Tol1 and *C. glutamicum* wild type cultivated in minimal medium with 100 mM glucose in presence or absence of methanol.

Gene ID	Gene name	Annotation	M-values (+ MeOH/ - MeOH) [a)]	
			Tol1	WT
cg0180	*maa*	Maltose O-acetyltransferase	1.0	n.d.
cg0607	-	Putative secreted protein	n.d.	1.2
cg0689	-	Hypothetical protein	1.1	n.d.
cg0697	-	Conserved hypothetical protein	n.d.	1.0
cg0762	*prpC2*	2-Methylcitrate synthase, involved in propionate catabolism	1.2	-0.6
cg0791	*pyc*	Pyruvate carboxylase	0.3	1.2
cg0792	-	Hypothetical protein	n.d.	1.2
cg0952	-	Putative integral membrane protein	-0.2	1.2
cg0953	-	Putative Na+/solute symporter, solute:sodium symporter (SSS) family	0.4	1.1
cg1291	-	Putative membrane protein	2.0	1.0
cg1327	-	Putative transcriptional regulator, Crp-family	1.2	n.d.
cg1328	-	Putative heavy-metal ion transporting P-type ATPase	1.2	n.d.
cg1392	-	Putative transcriptional regulator, HTH_3-family	1.2	n.d.
cg1484	-	Putative secreted protein	1.1	n.d.
cg1625	-	Hypothetical protein	1.7	1.6
cg1895	-	Putative secreted protein	1.3	n.d.
cg1917	-	Hypothetical protein	1.0	n.d.
cg2004	-	Conserved hypothetical protein	1.1	n.d.
cg2047	-	Putative secreted protein	n.d.	1.1
cg2226	-	Conserved hypothetical protein, Rossmann-fold nucleotide-binding protein involved in DNA uptake	1.2	n.d.
cg2500	-	Putative transcriptional regulator, ArsR-family	1.1	n.d.
cg2559	*aceB*	Malate synthase	0.7	2.9
cg2560	*aceA*	Isocitrate lyase	0.7	4.3
cg2846	*pstS*	ABC-type putative phosphate transporter, substrate-binding lipoprotein (TC 3.A.1.7.1)	2.8	n.d.
cg2962	-	Hypothetical protein, uncharacterized enzyme involved in biosynthesis of extracellular polysaccharides	2.2	n.d.
cg3047	*ackA*	Acetate kinase	0.6	2.6
cg3048	*pta*	Phosphate acetyltransferase	n.d.	2.1
cg3107	*adhA*	Alcohol dehydrogenase	1.5	1.7
cg3117	*cysX*	Ferredoxin-like protein, involved in electron-transfer	0.3	1.1
cg3192	-	Putative secreted or membrane protein	1.3	n.d.
cg3219	*ldh*	L-Lactate dehydrogenase	2.4	n.d.
cg3255	*uspA3*	Universal stress protein E	1.3	n.d.
cg3272	-	Putative membrane protein	1.6	n.d.

Table S2 (continued)

Gene ID	Gene name	Annotation	M-values (+ MeOH/ - MeOH) [a)]	
			Tol1	WT
cg3288	-	Hypothetical protein	n.d.	1.0
cg3303	-	Putative transcriptional regulator, PadR-family	2.5	n.d.
cg3327	*dps*	Putative starvation-induced DNA protecting protein	1.3	n.d.
cg3347	-	Putative secreted or membrane protein	1.0	n.d.
cg3402	-	Putative Hg2+ permease, MerTP-family	1.9	n.d.
ISCg2	-	ISCg2 (cg2854|cg2426|cg0226|cg3151|cg2354)	1.2	n.d.
cg0133	-	Putative secondary aminobenzoyl-glutamate transporter, AbgT-family	n.d.	-1.2
cg0134	-	Hydrolase, Ama/HipO/HyuC-family	n.d.	-1.1
cg0277	-	Putative secondary Na+:di-/tricarboxylate symporter, divalent anion:Na+ symporter (DASS) family	n.d.	-1.2
cg0466	-	Conserved putative secreted protein	n.d.	-1.1
cg0719	*crtYe*	C50 carotenoid epsilon cyclase	n.d.	-1.0
cg0771	*irp1*	ABC-type putative iron-siderophore transporter, substrate-binding lipoprotein	n.d.	-1.1
cg0890	-	Conserved hypothetical protein	n.d.	-1.0
cg1846	-	Putative transcriptional regulator, TetR-family	-1.0	-0.8
cg1847	-	Conserved hypothetical protein	-1.1	n.d.
cg1877	-	Lauroyl/myristoyl acyltransferase, lipid A biosynthesis	-1.3	n.d.
cg2235	*rplS*	50S ribosomal protein L19	n.d.	-1.2
cg2836	*sucD*	Succinate--CoA ligase (ADP-forming), alpha subunit	n.d.	-2.7
cg2837	*sucC*	Succinate--CoA ligase (ADP-forming), beta subunit	n.d.	-2.0
cg2911	-	ABC-type putative Mn/Zn transporter, substrate-binding lipoprotein	-2.8	n.d.
cg2912	-	ABC-type putative Mn/Zn transporter, ATPase subunit	-2.4	n.d.
cg2913	-	ABC-type putative Mn/Zn transporter, permease subunit	-1.9	n.d.
cg2949	-	Putative secreted protein	n.d.	-1.5
cg3035	-	Putative GCN5-related N-acetyltransferase	-1.4	n.d.
cg3214	-	Conserved hypothetical protein	n.d.	-1.1
cg3335	*mez*	Malate dehydrogenase (oxaloacetate-decarboxylating) (NADP(+))	n.d.	-1.7
cg3395	*proP*	Putative proline/betaine permease, MFS-type	n.d.	-1.8
cg3404	-	ABC-type putative iron(III) dicitrate transporter, substrate-binding lipoprotein	n.d.	-1.7
cg3430	-	Conserved hypothetical protein	-1.0	n.d.
cg3431	*rnpA*	Ribonuclease P	-2.0	-0.6

[a)] Dual logarithm of the average ratio of medians. Only genes regulated more than two-fold in at least one strain were considered.
n.d.) No significant result. Only results with a p-value (adjusted with the False Discovery Rate approach) of ≤ 0.05 were considered to be significant.
Two respectively three biological replicates were performed.

Table S3. Mutations detected in strain Tol1 and a control strain in comparison to the *C. glutamicum* wild type genome sequence NC_006958.1.

Nucleotide in WT		WT Δ*hpr* control [a)] mutation		WT Δ*hpr* control [a)] amino acid change	Tol1 mutation		Tol1 amino acid change	Gene
48014	G	SNP	T	T3K	SNP	T	T3K	cg0062
66717	T	Del	Δ	-	Del	Δ	-	cg0085
170052	C				SNP	T	A19V	cg0198
268952	G	Ins	G	-	Ins	G	-	cg0304
273896	A	SNP	G	-	SNP	G	-	cg0309
278749	A	SNP	T	-	SNP	T	-	intergenic
322774	C	Del	Δ	-	Del	Δ	-	intergenic
364913	A	SNP	C	E363D	SNP	C	E363D	cg0414
390756	G	Ins	G	-	Ins	G	-	intergenic
400383	G	Ins	G	-	Ins	G	-	intergenic
409175	G	SNP	T	A267D	SNP	T	A267D	cg0464
554148	G	Del	Δ	-	Del	Δ	-	intergenic
554257	G	Del	Δ	-	Del	Δ	-	intergenic
554305	G	Del	Δ	-	Del	Δ	-	intergenic
578120	G	Ins	G	-	Ins	G	-	intergenic
668739	C				SNP	T	A165T	cg0755
844090	A	SNP	G	-	SNP	G	-	cg0911
844495	C	SNP	G	G50R	SNP	G	G50R	cg0911
851860	G	Ins	G	-	Ins	G	-	intergenic
892384	C	SNP	G	P1748A	SNP	G	P1748A	cg0957
960473	G	Del	Δ	-	Del	Δ	-	intergenic
978319	A	SNP	C	K188N	SNP	C	K188N	cg1052
1035302	C				SNP	G	-	intergenic
1060070	A	Ins	A	-	Ins	A	-	intergenic
1111144	G	Ins	G	-	Ins	G	-	cg1206
1126684	A	SNP	G	C269R	SNP	G	C269R	cg1226
1143723	T				SNP	C	L328S	cg1245
1232173	A	SNP	C	-	SNP	C	-	cg1325
1344201	C	Ins	A	-	Ins	A	-	intergenic
1344201	C	Ins	A	-	Ins	A	-	intergenic
1714259	A	SNP	C	K186Q	SNP	C	K186Q	cg1819
1753385	C	SNP	G	D140E	SNP	G	D140E	cg1859
1753388	G	SNP	T	-	SNP	T	-	cg1859
1753595	G	Del	Δ	-	Del	Δ	-	intergenic
1763157	C	SNP	T	V275I	SNP	T	V275I	cg1869
1765670	A	SNP	T	V182E	SNP	T	V182E	cg1872
1765671	C	SNP	G	V182L	SNP	G	V182L	cg1872
1811091	C	SNP	T	-	SNP	T	-	intergenic
1963729	A				SNP	G	-	cg2069
1963843	C				SNP	T	-	cg2069
1963845	T				SNP	G	-	cg2069
1963857	G				SNP	A	-	cg2069
1963885	A				SNP	G	-	cg2069
1963900	A				SNP	T	-	cg2069
1963903	C				SNP	A	-	cg2069
1963908	C				SNP	A	V191F	cg2069
1964274	A				SNP	T	S69T	cg2069

Table S3 (continued)

Nucleotide in WT		WT Δ*hpr* control [a)] mutation		amino acid change	Tol1 mutation		amino acid change	Gene
1964279	A				SNP	T	F67Y	cg2069
1964298	T				SNP	C	K61E	cg2069
1964302	A				SNP	T	-	cg2069
1964305	C				SNP	T	-	cg2069
1964329	G				SNP	A	-	cg2069
1964338	A				SNP	G	-	cg2069
1964352	G				SNP	A	Q43*	cg2069
1964370	T				SNP	C	K37E	cg2069
1964395	T				SNP	C	-	cg2069
1964407	T				SNP	C	-	cg2069
1995296	G	SNP	A	-	SNP	A	-	intergenic
2002299	G	SNP	T	-	SNP	T	-	cg2111
2005932	C	Del	Δ	-	Del	Δ	-	intergenic
2012000	G	SNP	T	G75V	SNP	T	G75V	cg2118
2028992	A	SNP	G	S164P	SNP	G	S164P	cg2135
2030556	C	SNP	T	-	SNP	T	-	intergenic
2030620	C				SNP	A	-	intergenic
2055662	C	SNP	G	V31L	SNP	G	V31L	cg2167
2066532	C	SNP	G	R81P	SNP	G	R81P	cg2176
2096312	G				SNP	C	D67H	cg2204
2145398	G	SNP	T	P47T	SNP	T	P47T	cg2262
2166305	C	Ins	C	-	Ins	C	-	intergenic
2245932	T	SNP	C	-	SNP	C	-	intergenic
2245934	C	SNP	A	-	SNP	A	-	intergenic
2253306	A	SNP	G	-	SNP	G	-	cg2368
2327190	T	Del	Δ	-	Del	Δ	-	intergenic
2335863	G	Del	Δ	-	Del	Δ	-	intergenic
2339425	T	Ins	T	-	Ins	T	-	intergenic
2347748	A	SNP	G	-	SNP	G	-	cg2460
2354473	C				SNP	G	-	intergenic
2354476	C	SNP	T	-	SNP	T	-	intergenic
2354634	C	SNP	G	V219L	SNP	G	V219L	cg2467
2361038	A	SNP	C	K176N	SNP	C	K176N	cg2475
2361920	T	Del	Δ	-	Del	Δ	-	intergenic
2361925	T	Del	Δ	-	Del	Δ	-	intergenic
2441888	T	Del	Δ	-	Del	Δ	-	intergenic
2575624	A	SNP	G	F212S	SNP	G	F212S	cg2701
2584857	C	Ins	C	-	Ins	C	-	cg2712
2701531	G				SNP	A	Q342*	cg2840
2714349	G	Ins	C	-	Ins	C	-	intergenic
2816685	C	Ins	G	-	Ins	G	-	intergenic
2855585	G	SNP	A	R427Q	SNP	A	R427Q	cg3003

Table S3 (continued 2)

Nucleotide in WT		WT Δ*hpr* control [a)]			Tol1			Gene
		mutation		amino acid change	mutation		amino acid change	
2877096	A	SNP	G	S45P	SNP	G	S45P	cg3018
2913129	C	Ins	C	-	Ins	C	-	intergenic
2913144	T	Ins	T	-	Ins	T	-	intergenic
3028090	A	SNP	G	N9S	SNP	G	N9S	cg3170
3046767	C	SNP	T	V54I	SNP	T	V54I	cg3182
3051028	C	SNP	G	V351L	SNP	G	V351L	cg3187
3061630	T	Del	Δ	-	Del	Δ	-	intergenic
3099764	T				SNP	C	-	cg3237
3163180	C				SNP	T	-	intergenic
3185193	A	Del	Δ	-	Del	Δ	-	intergenic
3186651	A	SNP	C	E244D	SNP	C	E244D	cg3339
3219803	C	SNP	G	G137A	SNP	G	G137A	cg3370

[a)] The strain Δ*hpr* was used as a control, which carries an in-frame deletion of the gene *hpr.* This control strain is derived from the same wild type at the same time period as strain Tol1 and was used to exclude variations between the genome of the WT used here and the sequence deposited in the genome database. The deleted nucleotides of the *hpr* deletion are not listed.

* SNP led to a translational stop codon.

Table S4. Oligonucleotides used in this study.

Name	Sequence (5’→ 3’)[a]	Purpose
T0198 A	GGACTACCGACCAATGTTCTG	Base exchange in cg0198
T0198 B	CATCGTGGGTTCAGCC**A**CACGCATCCGCCTTAG	Base exchange in cg0198
T0198 C	CTAAGGCGGATGCGTG**T**GGCTGAACCCACGATG	Base exchange in cg0198
T0198 D	CATGAACGGCTCGAGCATG	Base exchange in cg0198
T0755 A	CCACCTTCTCGTGGTTGTTG	Base exchange in cg0755
T0755 B	CTTCGGCGAGACTTTC**A**CCAACCCACAGGCAGAC	Base exchange in cg0755
T0755 C	GTCTGCCTGTGGGTTGG**T**GAAAGTCTCGCCGAAG	Base exchange in cg0755
T0755 D	CGACAATTCCAATGCTGACCAGTGG	Base exchange in cg0755
T1245 A	CACGCTGGTAGGTAATTTCAGCTCAGTG	Base exchange in cg1245
T1245 B	GCCAATGTATCCC**G**AGACTGGGTACACATAG	Base exchange in cg1245
T1245 C	CTATGTGTACCCAGTCT**C**GGGATACATTGGC	Base exchange in cg1245
T1245 D	CAGTCATGGACATCGACGAACTAGC	Base exchange in cg1245
T2204 A	CGCATTCGCCTCTGACGTTG	Base exchange in cg2204
T2204 B	GTTGAGCAGGTCGATGT**G**GCCGAG	Base exchange in cg2204
T2204 C	CTCGGC**C**ACATCGACCTGCTCAAC	Base exchange in cg2204
T2204 D	CCAGTTTAGTTTGGCTACAGGGTGTG	Base exchange in cg2204
T2840 A	CTCGAAATGTTGTCGAGGC	Base exchange in cg2840
T2840 B	GCCCACAG**T**AGATCTCTAACCACCCAG	Base exchange in cg2840
T2840 C	CTGGGTGGTTAGAGATCT**A**CTGTGGGC	Base exchange in cg2840
T2840 D	GTATGCACGACATCTGGTCTG	Base exchange in cg2840

[a] Bold bases are different to the wild-type

Supplementary figures

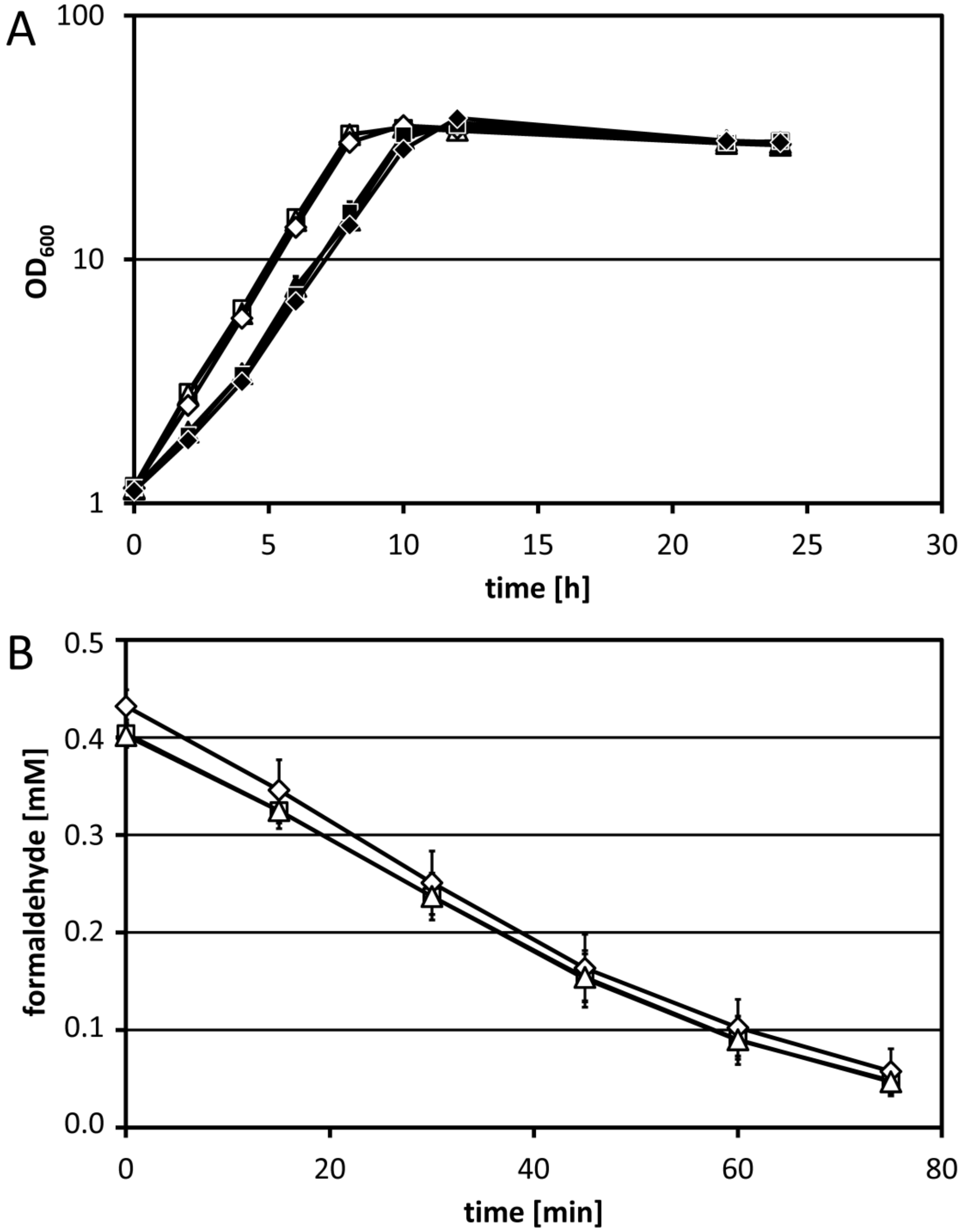

Figure S1. Growth in the presence of formaldehyde (A) and formaldehyde degradation (B). (A) Growth of *C. glutamicum* WT(pVWEx1) (diamonds) and strain Tol1 (triangles) in minimal medium with 100 mM glucose and without (open symbols) or with 1 mM (closed symbols) formaldehyde. (B) *In vivo* formaldehyde degradation assay of the strains mentioned above. Means and standard deviations of two independent cultures are shown.

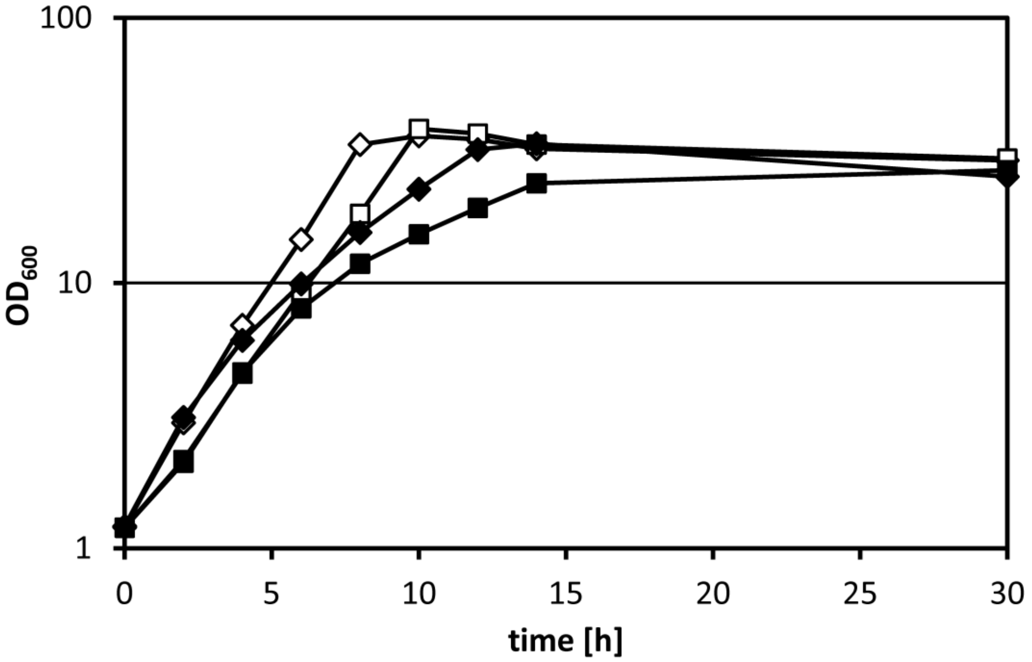

Figure S2. Growth of *C. glutamicum* wild type with propionate and methanol. *C. glutamicum* wild type was cultivated in minimal medium with 100 mM glucose (open symbols), supplemented with (squares) or without (diamonds) 0.5 g/l propionate. Addition of 120 mM methanol occurred after 2 h (closed symbols). Means and standard deviations of two independent cultures are shown.

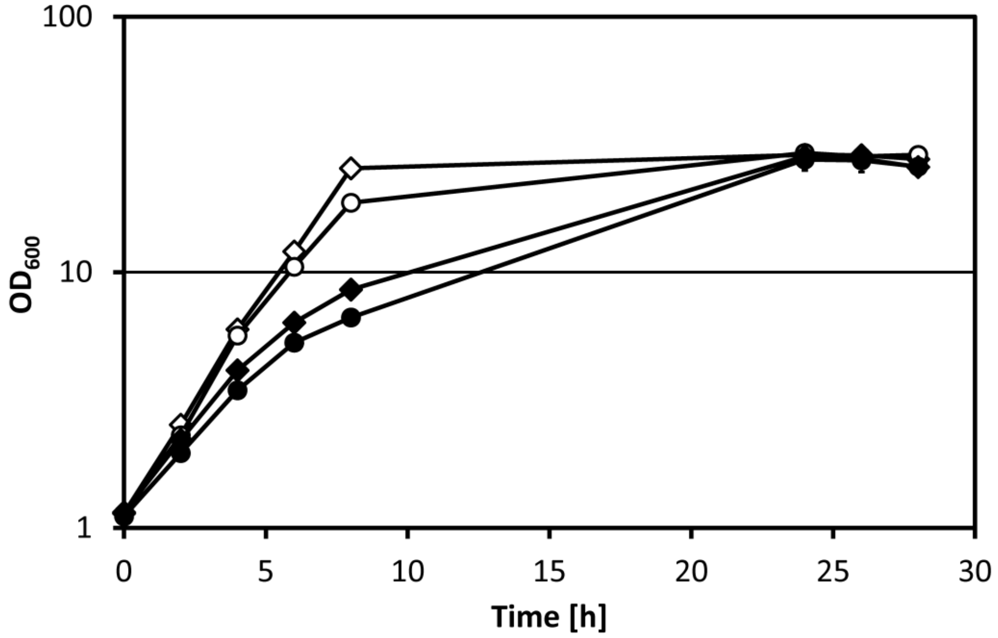

Figure S3. Growth of *C. glutamicum* Δ*ramA* with methanol. *C. glutamicum* wild type (diamonds) and the deletion mutant Δ*ramA* (circles) were cultivated in minimal medium with 100 mM glucose (open symbols) and additional 480 mM methanol (closed symbols). Means and standard deviations of two independent cultures are shown.

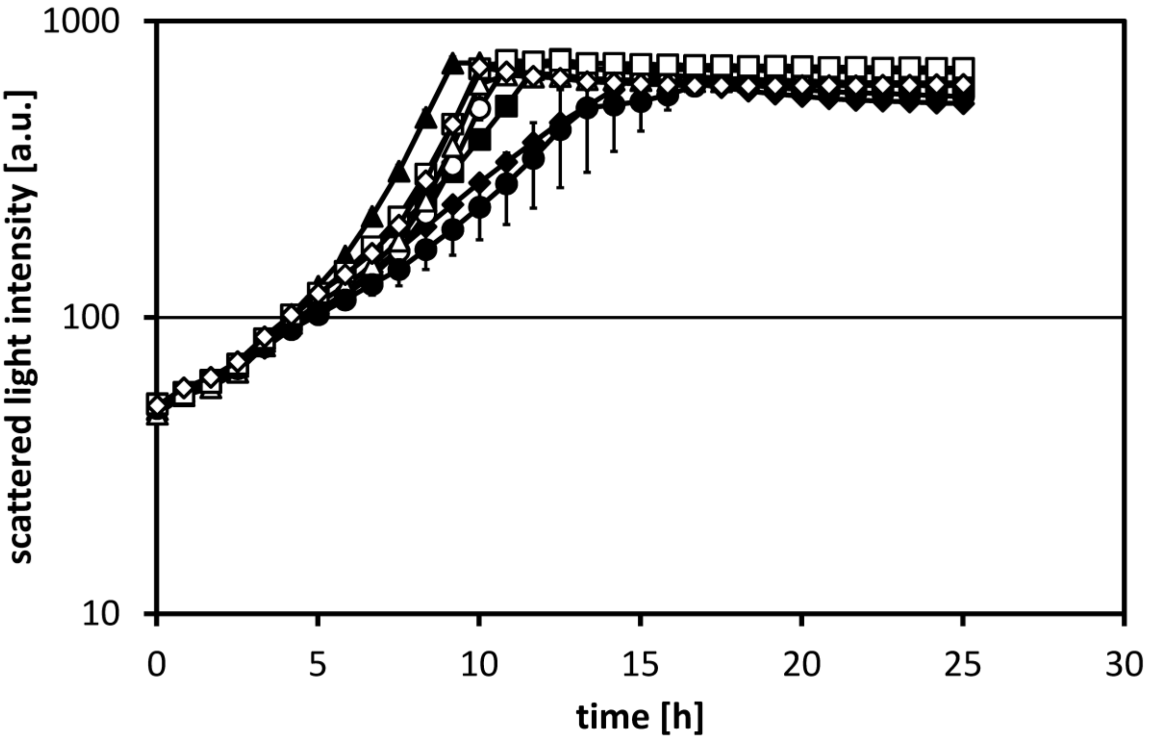

Figure S4. Growth of *C. glutamicum* Δ*cat* with methanol. Growth of *C. glutamicum* wild type (diamonds), Tol1 (triangles), T2840 (circles) and the deletion mutant Δ*cat* (squares) in minimal medium with 100 mM glucose (open symbols) and additional 240 mM methanol (closed symbols). Means and imprecision of two independent cultures are shown.

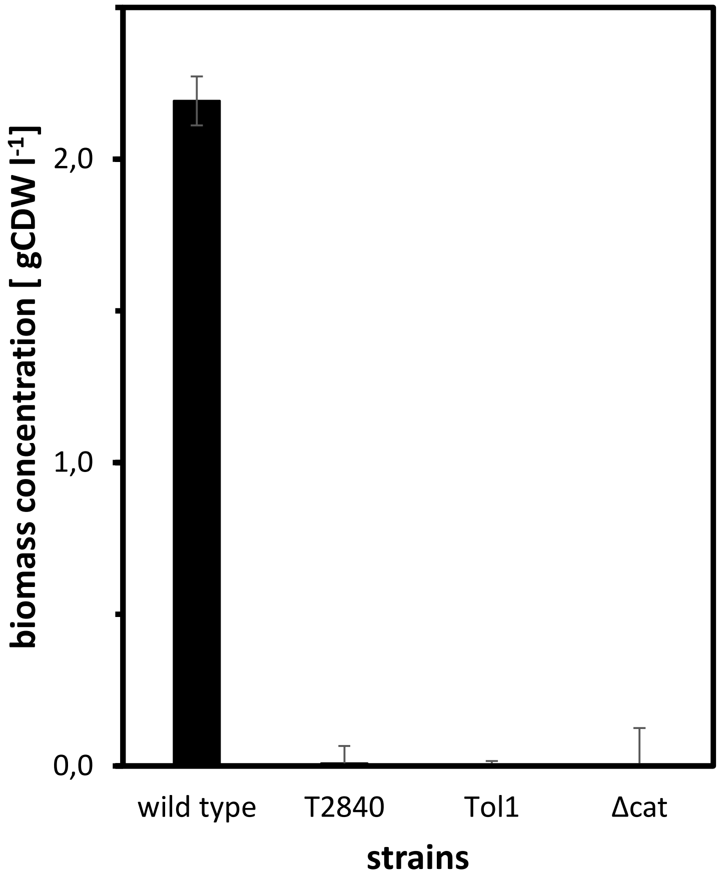

Figure S5. Biomass formation of *C. glutamicum* wild type, Tol1, T2840 and Δ*cat* in minimal medium with ethanol as sole carbon source. CgXII minimal medium with 1% ethanol was used. Means and standard deviations of three independent cultures are shown.
